# *Echinometra mathaei* and its ectocommensal shrimps: the role of sea urchin spinochrome pigments in the symbiotic association

**DOI:** 10.1038/s41598-018-36079-8

**Published:** 2018-12-03

**Authors:** Lola Brasseur, Guillaume Caulier, Gilles Lepoint, Pascal Gerbaux, Igor Eeckhaut

**Affiliations:** 1 Biology of Marine Organisms and Biomimetics unit, Research Institute for Biosciences, University of Mons – UMONS, 23 Place du Parc, B-7000 Mons, Belgium; 2 Laboratory of Oceanology, MARE Centre, University of Liège - ULG, B6c, 15 allée du six août, B-4000 Liège, Belgium; 3 Organic Synthesis and Mass Spectrometry Laboratory, Interdisciplinary Center for Mass Spectrometry, Research Institute for Biosciences, University of Mons – UMONS, 23 Place du Parc, B-7000 Mons, Belgium

## Abstract

*Tuleariocaris holthuisi* and *Arete indicus* are two ectocommensal shrimps closely associated with the tropical sea urchin *Echinometra mathaei*. This study provides a comparison of these two *E*. *mathaei* symbiotic crustaceans and particularly focuses on the relationship between *T*. *holthuisi* and its host’s pigments (i.e. spinochromes), and its dependency on its host. While all the analyses underline a close association between *A*. *indicus* and *E*. *mathaei*, they reveal a particularly close interaction between *T*. *holthuisi* and its host. Chemical analyses reveal that these shrimps present the same spinochrome composition as *E*. *mathaei*, and have similar colouration, allowing camouflage. Isotopic composition and pigment loss after host separation suggest that these pigments are certainly assimilated upon feeding on the urchin. Moreover, symbiont isolation experiments demonstrate the high dependency of *T*. *holthuisi* on its host and the importance of the host’s pigments on their survival capacity. Finally, some host recognition mechanisms are investigated for *T*. *holthuisi* and show the probable implication of spinochromes in host selection, through chemical recognition. Hence, all the results suggest the essential roles of spinochromes for *T*. *holthuisi*, which, in turn, suggests the potential implication of these pigments in the shrimps’ metabolism.

## Introduction

Symbioses are intimate associations between two heterospecific organisms (commonly called symbiont and host) that are generally classified into three categories: parasitism, commensalism and mutualism (these categories are themselves split into subclasses)^1–3^. According to the degree of dependence, symbioses are facultative or obligate, and the range of host-specificity of a symbiont varies significantly from one species to another. The evolutionary advantage of host-specificity for symbionts is to ensure they live in the appropriate habitat, but the symbiont can also be metabolically dependent on the host. This dependence is well known for many parasites (e.g. internal parasites, such as nematodes and trematodes) where the life cycle cannot be completed without the hosts^4^. It is also demonstrated in mutualists (e.g. the *Symbiodinium* algae living in corals) where nutrient exchange occurs^5^. The fine processes involved in the host-dependence of ectocommensals are, however, not well understood. It is apparent that, when doing a symbiont-host survey, some ectocommensals appear to be host-specific^6^ while others are more opportunistic^7^.

**Figure 1:**
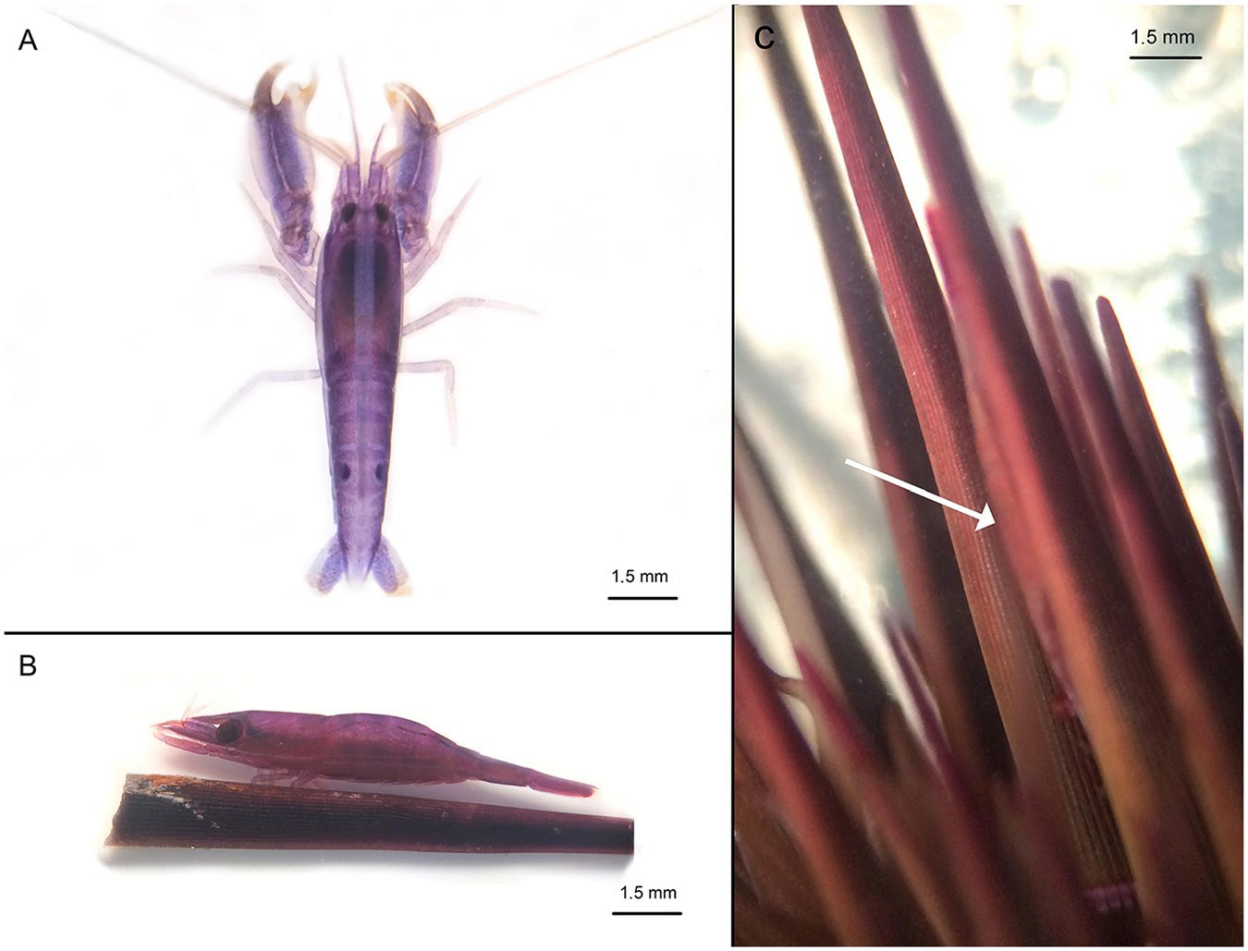
(**A**) *Arete indicus* Coutière, 1903; (**B**) *Tuleariocaris holthuisi* Hipeau-Jacquotte, 1965 on a sea urchin spine; (**C**) *Echinometra mathaei* (Blainville, 1825) hosting mimetic *T*. *holthuisi*.

Shrimps are involved in many marine symbioses with varied taxa^8^. Some shrimp families, like Pontoniine shrimps (Palaemonidae), have up to 60 to 70% of their representatives that live associated with marine organisms like corals, ascidians, gorgonians, sponges or echinoderms^9^. Among the echinoderms, the sea urchin *Echinometra mathaei* (Blainville, 1825), which is widespread in the Indo-Pacific Ocean, is the host for two crustacean symbionts: *Tuleariocaris holthuisi* Hipeau-Jacquotte, 1965 (Palaemonidae) (Fig. 1B,C) and *Arete indicus* Coutière, 1903 (Alpheidae) (Fig. 1A) (Cimino and Ghiselin 2001; Hipeau-Jacquotte 1965; Gherardi 1991). *A*. *indicus* is found on 21% of *E*. *mathaei* throughout the Eilat lagoon (Red Sea)^6^, however, they do not show any evidence of host tissue ingestion^6^. Omnivorous food habits have been reported in four other species of *Arete* associated with Japanese sea urchins^10^. *A*. *indicus* is able to recognise the odour produced by *E*. *mathaei*, and a conspecific sea urchin *Diadema setosum*, in their olfactometric systems^6^. Gherardi (1991) also explored the effects of the shrimps’ separation from the sea urchin host and found that *A*. *indicus* is affected by two weeks of isolation, with isolated shrimps being smaller (around 20%) and paler than the controls remaining on the host. *Tuleariocaris* is a small genus of four shrimp species, with the *T*. *holthuisi* type species described the first time in Toliara in Madagascar, where the field work of the present study took place. Unlike *A*. *indicus*, the symbiotic relations between *T*. *holthuisi* and *E*. *mathaei* have not yet been described, and, to date, only the taxonomy has been described for the *Tuleariocaris* species^11,12^.

The two symbiotic shrimps, *A*. *indicus* and *T*. *holthuisi* were common on the *E*. *mathaei* sampled in Toliara (Madagascar), and both had a dark colouration, similar to the most common *E*. *mathaei* morphotype. *T*. *holthuisi* is, however, much more mimetic, and can be visually confused with the spines of the sea urchin. The two species may be found simultaneously on the same individuals, raising the question of the differences in their respective ecological niche, as well as the question of potential competition between the two species. Moreover, it is presently unknown if the two symbionts feed on their host (parasitism) or if they are true commensalists. Carbon and nitrogen stable isotopes have recently been used to study the diets of various echinoderm invertebrate^13,14^ or vertebrate^15^ symbionts. These analyses revealed that this method is a powerful tool to determine the food sources that are assimilated over a longer period of life. This method differs from the analysis of the gut contents, which will only reflect recently ingested food. In particular, stable isotope analyses have been performed on a parasitic gastropod of *E*. *mathaei*, *Vexilla vexillum*, which grazes on the spines and the integument covering the tests of sea urchins^14,16^.

Chemodetection is a very common process completed by many symbiotic organisms to find their hosts, particularly when the symbiosis is obligatory and concerns a specific host. Evidence of host selections triggered by chemical sensing involving echinoderms as hosts have been shown for polychaetes living on asteroids/holothuroids^17,18^, shrimps associated with crinoids^19^, ophiuroids associated with other ophiuroids^20^, fishes^21,22^ and crabs^23^ associated with holothuroids, and bivalves^24^, gastropods^16^, crabs^25,26^, shrimps^6,27^ and fishes associated with echinoids^28^. While chemodetection has been proven to play a major role in host selection, it is only recently that it was demonstrated that the symbiotic Harlequin crab *Lissocarcinus orbicularis* is attracted by the saponins produced by its holothuroid hosts^23^. Saponins are also considered as toxic molecules protecting sea cucumbers against predators^29^. Echinoderms develop various molecules for their defence. In echinoids, the polyhydroxynaphthoquinones (PHNQ), also known as spinochromes or echinochromes^30–32^, provide sea urchins with their coloration, and are involved in their protection through antibacterial^33–35^, antioxidant^35–38^ and potential immune activities^39–42^.

The present paper aims, firstly, to analyse both the level of host-dependence developed by the two shrimp species, and the impact of forced isolation from their sea urchin hosts. Secondly, the “symbiotic addiction” (chemodetection, protective effect) of the shrimps towards the host spinochromes is also investigated.

## Results

### *In situ* shrimp population

In Toliara bay, the prevalence of infestation is similar for both symbionts: 36% of *E*. *mathaei* are infested by *T*. *holthuisi* (Fig. 1B,C) and 43% by *A*. *indicus* (Fig. 1A). In most cases, only one individual (both shrimp species included) was observed per sea urchin, with 15 to 20% infested with two shrimps, and between 10 and 15% with three or more shrimps.

### Analyses

#### Spinochrome analyses

The colour similarity between *E*. *mathaei* and their symbionts is high, particularly for *T*. *holthuisi* (Fig. 1C), rendering them difficult to detect visually. The presence of seven spinochromes were detected in the *E*. *mathaei*-conditioned water: three isomers of Spinochrome B and Spinochrome 252, and two isomers of Spinochrome A and Echinochrome A. The compositions were confirmed by accurate mass measurements (ppm < 10). Three spinochromes, Spinochrome B–Iso 2, Spinochrome B–Iso 3 and Spinochrome A–Iso 3, were not detected in the extracts of the *E*. *mathaei* tests and spines (Table 1) (Supplementary Fig. S1).

**Table 1:** Major PHNQ molecules (i.e. more than 5% of total PHNQ) detected in *E. Mathaei*, and PHNQ molecules detected in the conditioned water and in the symbionts of *E*. *mathaei*.

Phnq	Retention Time (min)	Mw (U)	Predicted Formula^a^	*E*. *mathaei*^b^	*E*. *mathaei*-Conditioned Water	*T*. *holthuisi*	*A*. *indicus*
Spinochrome B – Iso 1	2.75	222	C_10_H_6_0_6_	x	x	x	x
Spinochrome B – Iso 2	9.27	222	C_10_H_6_0_6_	—	x	—	x
Spinochrome B – Iso 3	4.06	222	C_10_H_6_0_6_	—	x	—	—
Spinochrome 252	3.72	252	C_11_H_8_O_7_	x	x	—	—
Spinochrome E	1.73	254	C_10_H_6_O_8_	x	—	x	—
Spinochrome A – Iso 2	7.95	264	C_12_H_8_O_7_	x	x	x	x
Spinochrome A – Iso 3	9.28	264	C_12_H_8_O_7_	—	x	—	—
Echinochrome A	6.69	266	C_12_H_10_O_7_	x	x	x	—
Spinochrome C	6.73	280	C_12_H_8_O_8_	x	—	x	—

^a^Based on accurate mass measurements. ^b^(^42,48^). Further details about isomers structure are described in Brasseur *et al*.^48^.

For the symbiotic shrimps, five spinochromes were detected within the extract of *T*. *holthuisi*, with confirmation by mass spectrometry (ppm < 10). Echinochrome A was the most abundant spinochrome, followed by Spinochrome A, Spinochrome E, Spinochrome B, and, finally, Spinochrome C (Table 1, Supplementary Fig. S1). For *A*. *indicus* extracts, three spinochromes were detected: Spinochrome A–Iso 2 and two isomers of Spinochrome B (Table 1, Supplementary Fig. S1). Spinochrome A–Iso 3 and Spinochrome B–Iso 2 and –Iso 3 were the only spinochromes that did not seem to be present in the host’s tests, but were present in the conditioned water. The compositions were confirmed by accurate mass measurements (ppm < 10) and checked with the literature^42^.

#### Symbionts’ diet

**Figure 2:**
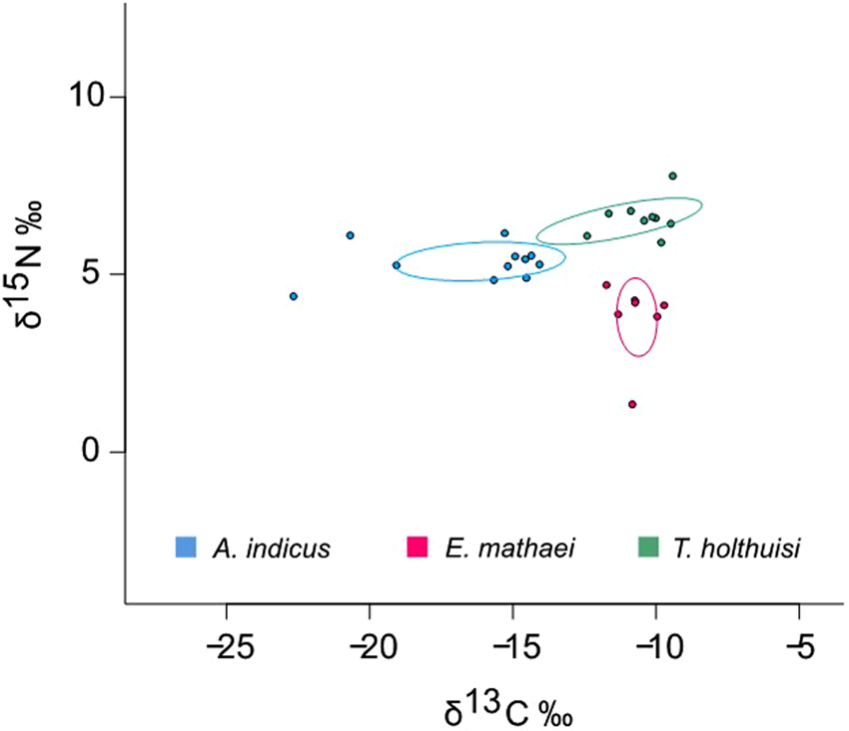
Values of δ^15^N and δ^13^C of *E*. *mathaei* symbionts and their potential food source, their host. Standard ellipse areas corrected for small samples SEAc (solid lines) are estimated for individuals of each species (symbols)^43^.

*Echinometra mathaei* has an average isotopic composition (±standard deviation) of 3.8 ± 1.1 for δ^15^N values, and −10.7 ± 0.7 for δ^13^C values (Fig. 2). For the isotopic signature of symbionts, *T*. *holthuisi* has a value of 6.5 ± 0.7 for δ^15^N and −11.3 ± 2.9 for δ^13^C. Finally, *A*. *indicus* has a value of 5.4 ± 0.6 for δ^15^N and −16.1 ± 3.0 for δ^13^C values. Statistical tests showed no significant differences between the δ^13^C composition of *T*. *holthuisi* and its host *E*. *mathaei* (Mann Whitney test, P > 0.05), but revealed significant statistical differences between their δ^15^N composition (Mann Whitney test, P < 0.0005) with a mean enrichment for *T*. *holthuisi* of 3‰. For *A*. *indicus*, statistical tests showed significant differences between the δ^13^C composition of *A*. *indicus* and its host *E*. *mathaei* (Mann Whitney test, P < 0.05) with a mean difference of 4.5‰, but revealed no significant differences between their δ^15^N composition (Mann Whitney test, P > 0.05).

The SEAc (Standard Ellipse Areas corrected for small sample)^43^ calculated for the *T*. *holthuisi* (4.8), *A*. *indicus* (5.6) and *E*. *mathaei* (2.9) populations showed distinct ecological niches for each species, with no overlap (Fig. 2).

### Experiments

#### Host chemodetection

**Figure 3:**
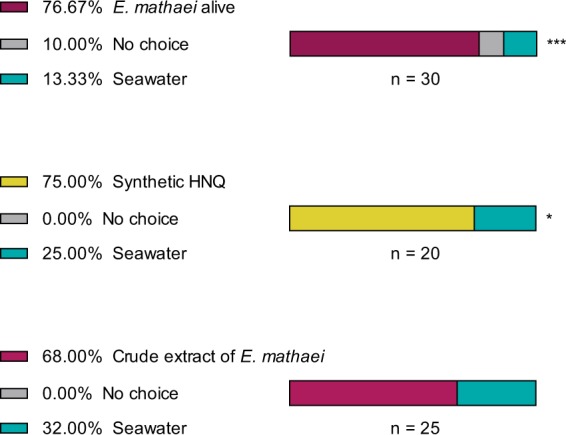
Behavioural experiments of chemical recognition on *T*. *holthuisi* for its host and potentially attractive compounds. ***p-value < 0.001; *p-value < 0.05; ^x^p-value > 0.05.

It was found that 66% of the tested *T*. *holthuisi* statistically preferred the seawater conditioned by their host than the control seawater (Binomial test, P < 0.001) (Fig. 3). Similarly, 75% of the tested shrimps preferred moving towards the seawater conditioned with the synthetic spinochromes (Binomial test, P < 0.05) rather than the control seawater. When offered the choice between the spinochrome crude extract of *E*. *mathaei* and the control seawater, the shrimps were not significantly attracted by the spinochrome crude extract (Binomial test, P > 0.05).

#### Depigmentation

**Figure 4:**
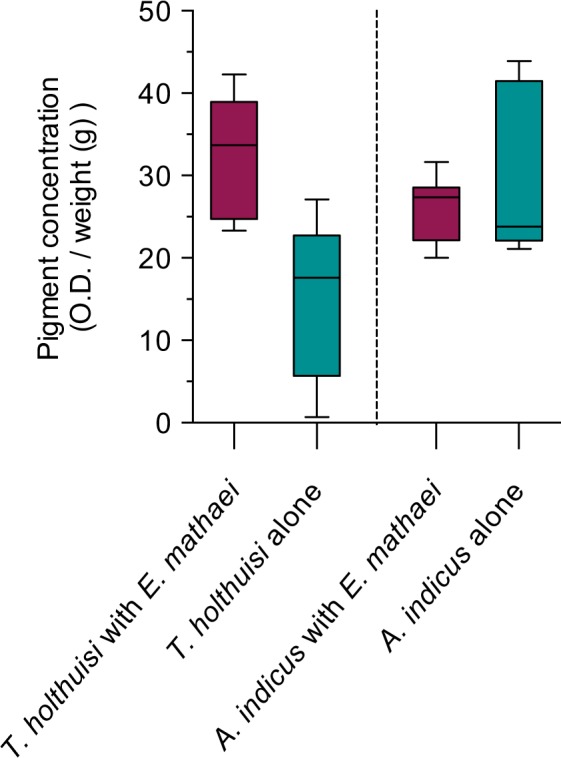
Pigment concentration in *E*. *mathaei* symbionts (O.D./weight (g)) with and without their hosts after 5 days. The boxplots include the minimum, lower quartile, median, upper quartile and maximum.

The spinochrome solution extracted from *T*. *holthuisi* living on *E*. *mathaei* showed an average absorbance at 450 nm (±standard deviation) of 31.98 ± 7.23, while the absorbance significantly decreased to 15.29 ± 9.10 after a five-day separation (Mann Whitney test, P < 0.0001) (Fig. 4). The spinochrome solution extracted from *A*. *indicus* living on *E*. *mathaei* showed an average absorbance (±standard deviation) of 25.86 ± 3.88. There was no significant decrease of absorbance after five days (30.19 ± 10.49) (Mann Whitney test, P > 0.05). Spinochrome concentrations in *T*. *holthuisi* and *A*. *indicus* were similar when they lived on their host (Mann Whitney test, P > 0.05) but were significantly lower for *T*. *holthuisi* than *A*. *indicus* when they were isolated (Mann Whitney test, P ≅ 0.01).

#### Shrimp survival

**Figure 5:**
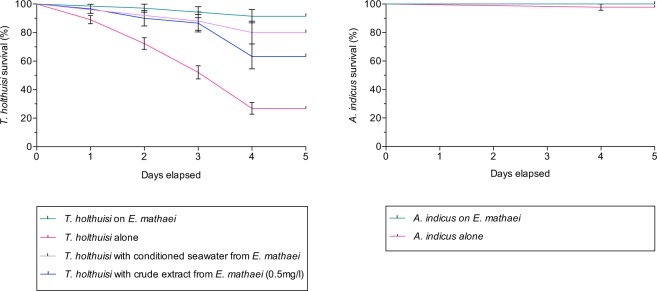
Survival curves of *T*. *holthuisi* and *A*. *indicus* according to different conditions. The data is expressed as the mean of living shrimps (± standard error).

The survival rate of *T*. *holthuisi* decreased rapidly when they were separated from their hosts (Fig. 5). In contrast, after five days, the survival rate for *T*. *holthuisi* individuals that were not separated from their hosts was 98%. The two survival curves are statistically different from one another (A Log-rank Mantel-Cox test, P = 0.0001).

The survival rate of *T*. *holthuisi* in *E*. *mathaei*-conditioned seawater after five days was significantly higher than when individuals were separated and placed in normal seawater (A Log-rank Mantel-Cox test, P ≅ 0.0001). There was no significant difference (A Log-rank Mantel-Cox test, P > 0.05) between the survival rate of *T*. *holthuisi* in *E*. *mathaei*-conditioned seawater and that of individuals that were not separated from their hosts.

The survival rate of *T*. *holthuisi* in seawater with spinochromes was statistically higher compared to the *T*. *holthuisi* separated and placed in normal seawater (A Log-rank Mantel-Cox test, P ≅ 0.0002). The survival rate of *T*. *holthuisi* in seawater with spinochromes was statistically similar to the survival rate of individuals with *E*. *mathaei*-conditioned seawater (A Log-rank Mantel-Cox test, P > 0.05), and was statistically different from the survival rate of individuals on their host (A Log-rank Mantel-Cox test, P ≅ 0.0002).

The survival rates of *A*. *indicus*, with or without the host, were respectively 100 and 99%. The survival rates were not statistically significant (A Log-rank Mantel-Cox test, P > 0.05).

## Discussion

Echinoderm recognition through chemodetection is performed by many ectocommensals, and is one of the main parameters that ensures the transgenerational sustainability of symbiotic associations^6,16,20,23,44,45^. Two ectocommensal symbioses associated with echinoderms and decapods had previously been investigated in order to identify the nature of the chemical signals allowing host selection. The saponins of sea cucumber hosts are kairomones that attract the symbiotic Harlequin crabs^23^, and anthraquinones extracted from crinoid hosts attract the symbiotic Stimpson’s Snapping Shrimp (Caulier *et al*., *submitted*). Our data reveal that various host spinochromes are found in the water surrounding sea urchins and that these molecules act as kairomones attracting the symbiotic shrimp *T*. *holthuisi*. This was partially shown for *A*. *indicus* in a previous study^6^, where it was found that 68% of shrimps were attracted by the crude extract of *E*. *mathaei*. It is possible that molecules other than spinochromes are present in the chemical cue and may disturb symbiont chemodetection, as shown in other recent behavioural experiments (data not published). However, according to previous studies, both saponins and spinochromes are known to act as chemical defence mechanisms for echinoderms: saponins are a toxic predator repellent^29,45^ and spinochromes are involved in antibacterial and antioxidant processes^34,35,37,42,46,47^. In both cases, this involves a remarkable co-evolutionary mechanism in which a host’s chemical defences are diverted by the symbionts to their own benefit. Sea urchin tests and spines contain different cocktails of spinochromes that may be species-specific. The species analysed in the present work, *E*. *mathaei*, possesses five spinochromes in their tests and spines, with a total of seven spinochromes observed in the water surrounding the sea urchins. The higher number of spinochromes seen in the water may be the result of UV isomerisation.

Spinochromes are kairomones allowing *T*. *holthuisi* to recognise their host, and they also allow the shrimp’s colour to perfectly match that of the sea urchin. In a recent study on *E*. *mathaei*^48^, six spinochromes were detected in the tests and spines of the sea urchin: Spinochrome B, Spinochrome E, Spinochrome A–Iso 2, Spinochrome 252, Echinochrome A and Spinochrome C (supplementary Table S1 and Fig. S1). In the present study it was observed that the *T*. *holthuisi* pigmentation is certainly due to the presence of spinochromes taken from the host: *T*. *holthuisi* possesses five spinochromes that are the same as *E*. *mathaei*, and the ^13^C/^12^C with the ^15^N/^14^N analyses suggest that the sea urchin is the main food source for the shrimp (meaning that shrimp spinochromes would be acquired during feeding). The ^13^C/^12^C ratio of *T*. *holthuisi* is similar to its host, with a higher ^15^N/^14^N ratio, which is typical of organisms that have extremely narrow food sources and, in the case of symbionts, that feed exclusively on their hosts. This was observed for a parasitic gastropod of *E*. *mathaei*, *Vexilla vexillum* which grazes on the spines and the integument covering the tests of sea urchins^16^. However, while *T*. *holthuisi* feed on their host, they do not seem to cause injuries, as there were no lesions observed on *E*. *mathaei* (personal observation). Thus, although *T*. *holthuisi* could be considered a parasite, it is estimated that the impact on the host’s fitness is limited, and that it is more appropriate to consider this type of symbiosis as commensal^1^.

In comparison, *Arete indicus* are less mimetic and the shrimps can be easily seen on their hosts. *A*. *indicus* contains three spinochromes in common with *E*. *mathaei*, but stable isotope analyses did not show host-dependent feeding: the large standard deviation observed with ^13^C/^12^C values indicates that their food sources are diverse, and the few overlaps with *T*. *holthuisi* suggest a different diet. *A*. *indicus* could feed on *E*. *mathaei* (i.e. in order to ingest naphthoquinones), but not exclusively, and various other food items are probably also eaten by this species (benthic organisms or organic matter).

The analyses also demonstrate that besides spinochromes being involved in *T*. *holthuisi* host recognition, and the fact that they provide protection and efficient mimicry, the spinochromes are also involved in much more intimate physiological processes that are essential for the shrimps’ survival. When isolated from its host, *T*. *holthuisi* suffers from depigmentation, and most individuals die within five days following host separation. Depigmentation is evident in host-separated *T*. *holthuisi*, in comparison with the results obtained for the least host-dependant shrimp, *A*. *indicus*. Dependence of *T*. *holthuisi* on *E*. *mathaei* is also obvious when the survival rate of separated *T*. *holthuisi* is compared to that of non-separated individuals or to separated *A*. *indicus*. The survival rate of separated *T*. *holthuisi* is, moreover, significantly increased when individuals are incubated in *E*. *mathaei*-conditioned seawater, or in sea urchin extracted spinochromes. These results suggest that *E*. *mathaei* is not only a “house” for *T*. *holthuisi*, or its unique food source, but that spinochromes are essential for *T*. *holthuisi* survival.

Three hypotheses may be formulated to explain *T*. *holthuisi* death in the absence of spinochromes: (i) spinochromes (and other dissolved organic molecules) are the only food source on which the shrimps rely, (ii) the antibacterial and antioxidant properties of spinochromes are essential to protecting the shrimps, or (iii) spinochromes are integral or promote physiological pathways essential for the shrimps’ metabolism. The latter hypothesis may be the most plausible, as spinochromes would almost certainly not provide the necessary nutrients, and it seems unlikely that bacteria or the UV effect of the sun has influenced the survival rates in the experiments (the seawater was filtered and assays were performed in laboratory conditions). The spinochrome “addiction” of *T*. *holthuisi* was also observed when the shrimps were separated from their host and placed in the same aquarium as the parasitic gastropod *V*. *vexillum*. Under these conditions, it was observed that the shrimps were highly attracted to the gastropod which had ingested spinochromes by feeding on sea urchins.

Evolution has brought about various degrees of host dependence amongst symbionts, some will be loosely dependent, such as in the case of *A*. *indicus*, whereas others will be highly dependent, as seen in *T*. *holthuisi*. The first group present the advantage of not being exclusively reliant on one host species, and can probably survive to the extinction of a potential host. Conversely, they are certainly less protected, as they are less mimetic and can also use various food sources in their diet. Host-dependent commensals benefit from an “all in one” home, but their evolution and survival are entirely linked to that of their hosts.

## Material and Methods

### Samples

The sea urchin *Echinometra mathaei* (Blainville, 1825) and its symbionts, *Tuleariocaris holthuisi* Hipeau-Jacquotte, 1965 and *Arete indicus* Coutière, 1903, were collected by scuba diving on the flat of the Great Reef of Toliara, Madagascar (23°23′34″S, 43°38′47″E) in November 2015 and November 2016.

*E*. *mathaei* (n = 105) were observed *in situ* and the number of shrimps of the two species per sea urchin, and the number of infested individuals, were recorded. *T*. *holthuisi* (n = 350), *A*. *indicus* (*n* = *130*) and *E*. *mathaei* (n = 50) were sampled for the behavioural experiments and spinochrome extractions.

### Analyses

#### Extraction and characterisation of spinochromes

The spinochrome cocktails^48^ that may be present in water conditioned by *E*. *mathaei* and those that may be present in *T*. *holthuisi* and *A*. *indicus* were analysed. For the analysis of the shrimp spinochromes, 30 fresh individuals of each species were dried for 2 hours at 90 °C, pooled and their spinochromes extracted as mentioned below. For the analysis of the spinochromes present in *E*. *mathaei*-conditioned seawater, three *E*. *mathaei* were placed in three tanks (14 cm × 18 cm × 15 cm) of 1 l each and filled with 1 µm filtered seawater at 25 °C for 24 h. The water was evaporated to dryness at low pressure at 60 °C using a rotary evaporator (Laborota 4001 efficient, Heidolph, Germany).

The spinochrome extraction started with 1 hour of maceration in 6M HCl (10 ml/5 g of samples) before being filtrated under vacuum with a Buchner flask. The solution was partitioned three times with diethyl ether (v/v). The diethyl ether phases were recovered, pooled and partitioned three times with NaCl 5% solution (v/v). Then, the final ethereal phase was recovered and evaporated to dryness at low pressure at 60 °C using a rotary evaporator (Laborota 4001 efficient, Heidolph, Germany), dissolved in 80% methanol, and centrifuged at 10,000 g for 10 minutes. Finally, the supernatant was recovered for analysis.

A Waters Alliance 2695 liquid chromatography device (HPLC) was used to separate the pigments. The system comprises a quaternary pump, a vacuum degasser and an autosampler. The chromatography was performed on a reversed phase column (Kinetex® 5 µm Biphenyl 100 Å, 50 × 4.6 mm, Phenomenex) at 30 °C, with an injected sample volume of 25 µl and a constant flow (1.25 ml/min) of a gradient of eluent A (Water, 0.1% formic acid) and eluent B (Acetonitrile) (supplementary Table S1).

The HPLC analysis device was coupled with a mass spectrometer, allowing the pigments to be determined. The mass spectrometry spectra were obtained on a Waters Quattro Ultima by scanning between m/z 50 and 1500 using an Electrospray ionisation (ESI) source operated in the negative ionisation mode. The ESI conditions were as follows: capillary voltage of 3.1 kV, cone voltage of 40 V, source temperature at 120 °C and desolvation temperature at 300 °C. Dry nitrogen was used as the ESI gas with a flow rate of 50 l/h for the gas cone and 500 l/h for the desolvation gas.

The accurate mass measurements and molecular formula of PHNQ ion predictions were performed on a Waters Q-ToF Premier using an Electrospray ionisation source in the negative ionisation mode, by scanning between m/z 50 and 600 with scan durations of 1 s and an inter-scan time of 0.1 s. The ESI conditions were as follows: capillary voltage of 3.1 kV, cone voltage of 40 V, source temperature at 120 °C and desolvation temperature 300 °C. Dry nitrogen was used as the ESI gas with a flow rate of 50 l/h for the gas cone and 600 l/h for the desolvation gas. The mass spectrometer was equipped with a lockspray setup to obtain high mass accuracy of PHNQ ions. Sodium iodide was used as a reference sample with m/z 126.9045 as the lock mass (iodide anion). Mass spectra analyses were performed on MassLynx 4.1. mass spectrometry software (Waters, Milford, MA, USA) and compared to the literature^42^. PHNQ were drawn using “ChemDraw 15.0.0.106” (PerkinElmer Informatics. Inc.) software and annotated with the “Affinity Designer” software.

#### Host-dependent feeding

The diets of symbiotic organisms can be diversified; they can be dependent on the host if they feed on their tissues or divert food caught by the hosts^14^. As spinochromes are partly in the body wall of sea urchins, it was necessary to determine whether *T*. *holthuisi* and *A*. *indicus* fed on *E*. *mathaei* tissue. For this, 10 *T*. *holthuisi*, 10 *A*. *indicus* and 10 *E*. *mathaei* were collected to perform stable isotope analyses. Integument tissues were scraped from the sea urchins. The samples were dried at 60 °C for 24 h before being crushed. All samples were acidified using an HCL fumigation technique (fuming HCL, 37%, Merck) for 48 h in order to remove the calcium carbonate. Indeed, inorganic carbon does not reflect the isotopic composition of an animal’s diet. Isotopic ratios and elemental content measurements were performed using an isotopic ratio mass spectrometer (IsoPrime100, Isoprime, UK) interfaced in continuous flow with an elemental analyser (vario MICRO cube, Elementar, Germany). Isotope ratios for C and N were reported conventionally^49^ in per mil (‰) using standard delta (*δ*) notation relative to their respective international standards, Vienna-Pee Dee Belemnite (V-PDB) and atmospheric N_2_:

1 $$\delta X=(\frac{{R}_{sample}-{R}_{standard}}{{R}_{standard}})\times {10}^{3}(\textperthousand )$$

where *X* = ^13^C or ^15^N, *R* = ^13^C/^12^C or ^15^N/^14^N. Analytical precision was assessed by procedural blanks, internal replicates (i.e., glycine, in-house crustacean and seagrass reference material) and isotopic certified material: sucrose (IAEA-C6; *δ*^13^C = −10.8 ± 0.3‰) and ammonium sulfate (IAEA-N2; *δ*^15^N = 20.3 ± 0.3‰), obtained from the International Atomic Energy Agency (IAEA, Vienna, Austria). Standard deviations on replicated measurements presented hereafter were 0.1‰ for *δ*^13^C and 0.2‰ for *δ*^15^N. Neither chemical lipid extractions nor *a posteriori* lipid corrections were performed, due to the often limited relevance of *a posteriori* corrections for aquatic invertebrates containing high proportions of chitin in addition to lipids and proteins^50^. The isotopic compositions were analysed with the SIBER package compared using the Mann Whitney test with the “Prism 6” (GraphPad) software. The isotopic ecological niches were compared using the Stable Isotope Bayesian Ellipses package (SIBER) in R version 2.2.2.

### Host selection and symbiont isolation

#### Host chemodetection

The experiments were carried out to study host chemical recognition using *T*. *holthuisi* with three differently conditioned seawaters, each as a potential chemical stimulus. First, the test was performed on 30 individuals with conditioned seawater. Conditioned seawater was obtained by submerging two individuals of *E*. *mathaei* per litre of filtered (1 µm) seawater for 2 hours. Secondly, 20 individuals were tested with synthetic pure molecules: 2-hydroxynaphtoquinone (Sigma Aldrich St. Louis, MO, USA) at a concentration of 0.1 g/l of seawater. This concentration was chosen in accordance with Caulier *et al*^23^., and recent behavioural experiments using another symbiosis between an urchin and a crab (non-published data). Lower concentrations were also partially tested (1 mg/l, 100 µg/l) and provided similar results. Finally, 25 individuals were tested with the *E*. *mathaei* crude extract at a concentration of 1 mg per litre of seawater. The experiments were performed with a Davenport olfactometer^23^. The latter is composed of a Y-shaped glass tube of 2 cm diameter, 20 cm length and a 10 cm region where the paired branches are connected to two opaque tanks of 1 l each. One tank was filled with the test seawater and the second contained the control seawater. Both experimental seawaters were filtered and controlled to 25 °C and 35 for salinity, and filtered at 1 µm. The seawaters flowed from the two tanks through the Y-tube and were evacuated at the base of the unpaired branch at a speed of 2-3 cm/s. Fluorescein was used prior to the experiments in order to control the flow turbulence inside the olfactometer. The tested shrimp was first introduced at the base of the unpaired branch. If the shrimp was stimulated, it moved into the unpaired branch to the junction and potentially chose one of the paired branches. The orientation of the shrimp was recorded. The position of the test seawater and control seawater was inverted after every 5 replicates after a complete washing of the Davenport system. The shrimps were considered to have made a choice when they entered one of the two tanks. After more than 5 minutes of testing without any movement, the trials were considered as “No choice”. Each shrimp was tested only once. The shrimps’ orientation preferences were analysed using a binomial test relative to a random distribution (50/50) with the “Prism 6” (GraphPad) software.

#### Depigmentation

Some observations during the survival tests suggested a pigmentation loss after the shrimps were separated from their host. In order to quantify this depigmentation, a comparison test was performed under two conditions:

(i) Five individuals from each shrimp species were placed on one *E*. *mathaei* (number of tanks = 2; number of shrimps = 10). The seawater was replaced every day with fresh filtered (1 µm) seawater.
(ii) Five individuals from each shrimp species were placed without their host (number of tanks = 3 for *T*. *holthuisi* and 1 for *A*. *indicus*; number of shrimps = 15 for *T*. *holthuisi* and 5 for *A*. *indicus*). The seawater was replaced every day with fresh filtered (1 µm) seawater.

All the experiments were performed for five days in tanks of 14 × 18 × 15 cm containing 1 l of filtered (1 µm) seawater at 25 °C. Live shrimps were collected at the end of the tests and dried at 90 °C for 2 hours. Each shrimp was then weighed, placed in 1 ml of 100% ethanol to extract the pigments and stored in the dark at 5 °C before being measured. Later, the absorbance of the ethanol solution was measured at 450 nm cm^−1^. Pigment concentrations are expressed as optical density (O.D.) divided by dried weight. The pigment concentrations were compared using the Mann Whitney test with the “Prism 6” (GraphPad) software.

#### Shrimp survival

*Tuleariocaris holthuisi* survival times and rates (i.e. number of shrimps that survived per day) were recorded under four treatments:

(i) Five individuals were placed on one *E*. *mathaei* (number of replicates = 7; number of shrimps = 35). The seawater was replaced every day with fresh filtered (1 µm) seawater.
(ii) Five individuals were placed without their host (number of replicates = 25; number of shrimps = 120). The seawater was replaced every day with fresh filtered (1 µm) seawater.
(iii) Five individuals were incubated in *E*. *mathaei-*conditioned sea water (number of replicates = 5; number of shrimps = 25). The seawater was replaced everyday with fresh filtered (1 µm) seawater conditioned for 24 h by one *E*. *mathaei*.
(iv) Five individuals were incubated in sea water with 0.5 mg/l of *E*. *mathaei* spinochromes crude extract from tests and spines (number of replicates = 6; number of shrimps = 30). The seawater was replaced every day with fresh filtered seawater (1 µm) including 0.5 mg/l of host crude extract.

As the host-dependence shown by *Arete indicus* was much lower, the survival times and rates were only estimated under the first and second conditions:

(i) Five individuals were placed on one *E*. *mathaei* in the same conditions as the *T*. *holthuisi* experiment (number of replicates = 7; number of shrimps = 35)
(ii) Five individuals without their host were placed in the same conditions as the *T*. *holthuisi* experiment (number of replicates = 8; number of shrimps = 40).

All the experiments were performed in tanks of 14 × 18 × 15 cm containing 1 l of filtered seawater at 25 °C. The number of live shrimps was verified every day at the same time for five days. The survival curves were calculated with survival analysis tools on the “Prism 6” (GraphPad) software. Their comparisons were performed with the Log-rank (Mantel-Cox) test.

### Ethics Statement

The animals used in our experiments were maintained and treated in compliance with the guidelines specified by the Belgian Ministry of Trade and Agriculture.

## Electronic supplementary material

Figure S1
