# Supplementary material for: Echinometra mathaei and its ectocommensal shrimps: the role of sea urchin spinochrome pigments in the symbiotic association
Source: Sci Rep. 2018 Dec 3;8:17540. doi: 10.1038/s41598-018-36079-8 (PMC6277410; doi:10.1038/s41598-018-36079-8)

Supplementary information

Echinometra mathaei and its ectocommensal shrimps: the role of sea urchin spinochrome pigments in the symbiotic association

Lola Brasseur[1†*], Guillaume Caulier[1†*], Gilles Lepoint[2], Pascal Gerbaux[3] and Igor Eeckhaut[1†]

Table S1 Gradient timetable used for the HPLC

TIME (MINUTES)	ELUENT A (%)	ELUENT B (%)	CURVE
00	80	20	Equilibration
00 → 15	80 → 50	20 → 50	Linear gradient
15 → 16	50 → 80	50 → 20	Linear gradient
16 → 18	80	20	Isocratic

Figure S1. Hypothetical structures of major *E. mathaei* pigments (i.e. more than 5% of total PHNQ content) identified in tests and spines. Further details about isomers structure are described in Brasseur et al. (2018). [51]

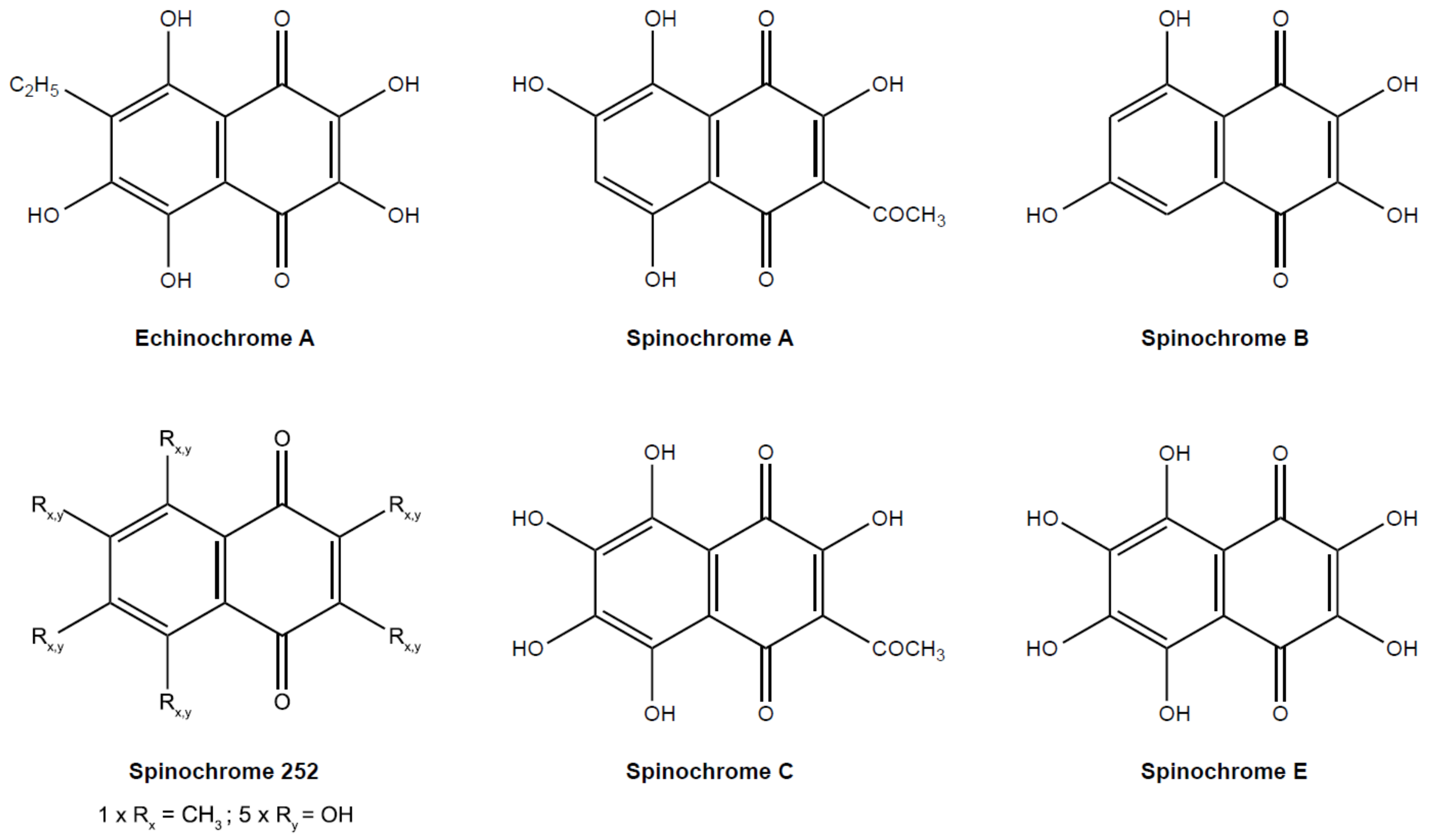

Supplement: Supplementary file 1 — Figure S1 [file 41598_2018_36079_MOESM1_ESM.pdf]
